# The role of physiotherapy in fibromyalgia: Current and future perspectives

**DOI:** 10.3389/fphys.2022.968292

**Published:** 2022-08-16

**Authors:** Mateus Dias Antunes, Amélia Pasqual Marques

**Affiliations:** Department of Physiotherapy, Rehabilitation Sciences Program, Speech-Language Pathology and Audiology and Occupational Therapy, Faculty of Medicine, University of São Paulo, São Paulo, Brazil

**Keywords:** fibromyalgia, rheumatology, physical therapy modalities (MeSH), rehabilitation, health promotion

## Abstract

Fibromyalgia is a chronic pain condition characterized by generalized musculoskeletal pain, hyperalgesia and allodynia, commonly associated with other symptoms such as fatigue, poor sleep quality, anxiety and depression. The clinical manifestations of this rheumatic disease have significant psychosocial and economic repercussions, with a substantial impact on health status, quality of life and social activities. Currently, recommendations for the management of fibromyalgia include patient education and non-pharmacological interventions, and among the indicated treatments, clinical guidelines include several physiotherapeutic resources, essential for individuals affected by this syndrome. Research in the physiotherapy field has demonstrated its effectiveness, but there is a need to update the literature. This study aims to identify the effectiveness of physiotherapy in the treatment of individuals with fibromyalgia. We performed a literature review looking for articles dated from March 2012 to March 2022 using the terms “fibromyalgia”, “physiotherapy”, “physical therapy”, “rehabilitation” in different languages in various databases and their main information was read and collected and presented in a descriptive way. The effects of physiotherapy interventions are summarized in order to provide a reference for future research and clinical application. Research on non-pharmacological physiotherapy-oriented treatments has grown in recent years as an alternative for fibromyalgia treatment. This review allows fibromyalgia patients to receive appropriate physical therapy interventions to promote their health.

## 1 Introduction

Fibromyalgia is a chronic pain condition characterized by generalized musculoskeletal pain, hyperalgesia and allodynia, commonly associated with other symptoms such as fatigue, poor sleep quality, anxiety and depression (Garrido-Ardila et al., 2021). The clinical manifestations of this rheumatic disease have significant psychosocial and economic repercussions, with a substantial impact on health status, quality of life and social activities (Borchers and Gershwin, 2015). The prevalence of this disease ranges from 0.2 to 6.6% in the general population and is mostly seen in women between the ages of 20–50 (Marques et al., 2017), causing disability with high direct (e.g., drug therapy and health care) and indirect costs (e.g., lost productivity) (Mascarenhas et al., 2021).

The cause and pathogenic process of fibromyalgia are not fully understood. Genetic and environmental factors and peripheral and central mechanisms are considered to be involved in causing widespread pain and pain sensitivity in individuals with fibromyalgia (Uruş et al., 2021).

As such, diagnosis is based on the clinical criteria described by the American College of Rheumatology and have been revised in recent years (Wolfe et al., 1990; Wolfe et al., 2011; Wolfe et al., 2016). In 1990, the American College of Rheumatology created the fibromyalgia classification criteria to aid in the diagnosis and standardize patients for clinical trials. This criterion is based on the presence of diffuse pain present for at least 3 months associated with 11 tender points (the palpation of the points was removed). However, in 2011, these criteria were revised to encompass other important aspects of fibromyalgia, such as fatigue, unrefreshing sleep, cognitive symptoms, headache, depression, and abdominal pain. In 2016, a revision of the 2010/2011 criteria was proposed to correct classification errors observed in patients with regional pain, a complementary criterion of diffuse pain was added (Wolfe et al., 1990; Wolfe et al., 2010; Wolfe et al., 2011; Wolfe et al., 2014; Wolfe et al., 2016).

Current recommendations highlight that primary treatment should include patient education and non-pharmacological interventions (Macfarlane et al., 2017; Kundakci et al., 2021). In addition, non-pharmacological treatments are recommended to improve symptoms related to pain, physical function and quality of life. Among conservative treatments, clinical guidelines include non-pharmacological therapies such as exercise therapy, mind-body therapies, patient education, manual therapy, needle therapies, balneotherapy, among others (Fitzcharles et al., 2013; Macfarlane et al., 2017; Garijo et al., 2022).

It is important for researchers to show the role of physiotherapy in individuals with fibromyalgia through current information as it contributes to improving clinical practice and scientific research. The present study summarizes the relevance of physiotherapy in the treatment of fibromyalgia patients to provide a reference for future research and clinical application.

### 2 Literature search

The terms fibromyalgia AND (physiotherapy OR “physical therapy” OR rehabilitation) were used as keywords in English, Portuguese and Spanish. The searches were not limited only to titles and abstracts, we chose to leave them free, appearing in all fields of the articles. In addition, some specific physical therapy interventions were sought manually. Searches were conducted on the Scientific Electronic Library Online (SciELO), Latin American and Caribbean Literature in Health Sciences (LILACS), Medical Literature Analysis and Retrieval System Online (MEDLINE), Scopus, Web of Knowledge ISI, Physiotherapy Evidence Database (PEDro), Excerpt Medical Database (Embase), Cumulative Index to Nursing and Allied Health Literature (CINAHL), Cochrane Library and SPORTDiscus for articles dated from March 2012 to March 2022. In addition, in order not to leave relevant articles out of the searches, we also searched other databases for articles related to the objective of the study. The articles were searched in all the indicated databases and then the main information was read and collected.

### 3 Physiotherapy in fibromyalgia

The different physical therapy modalities contribute to improving the quality of life of individuals with fibromyalgia (Melo et al., 2020). Below are described different physiotherapy modalities for the treatment of fibromyalgia.

### 3.1 Physiotherapy modalities for treating fibromyalgia

#### 3.1.1 Exercise

Fibromyalgia patients are generally intolerant to physical activity and tend to lead a sedentary lifestyle, increasing the risk of additional morbidity (Bidonde et al., 2017). Physical exercise is an important component in the treatment of fibromyalgia and given that its impact on psychological well-being is well established, several studies point to it as a means of reducing medication. In addition, individuals who engage in regular physical exercise exhibit better well-being compared to their sedentary counterparts, who share a negative self-perception of health. Another benefit of physical exercise is its effectiveness in reducing pain and fatigue. People with fibromyalgia have lower aerobic endurance and muscle strength, which can limit their ability to perform activities of daily living (Sauch Valmaña et al., 2020).

The European Alliance of Associations for Rheumatology (EULAR) strongly recommends exercise, mainly due to its effect on pain, physical function and well-being, availability, relatively low cost, and lack of safety concerns. Furthermore, EULAR underscores that there is consistent evidence in regard to aerobic and strengthening exercises, but insufficient data to suggest superiority of one over the other; and land and water exercises appear equally effective (Macfarlane et al., 2017).

Aerobic exercise can be performed without the need for suitable equipment or a proper environment, making it an affordable form of exercise for individuals with fibromyalgia. A Cochrane review (Bidonde et al., 2017) showed that aerobic exercise can improve quality of life, pain, stiffness and physical function compared to a control group, but does not appear to improve fatigue. The literature also reports that aerobic exercise is associated with reduced anxiety in adults with fibromyalgia. These findings support current knowledge and understanding of the role of aerobic-only exercises in the treatment of this disease. These exercises appear to be well tolerated and can be integrated into the treatment of adults with this condition. Thus, it is suggested that individuals with fibromyalgia can engage in simple and accessible physical activities without exacerbating pain and other symptoms, with an emphasis on aerobic training interventions such as walking, which seems well tolerated. As such, it makes sense to encourage walking since it is a free accessible activity (Bidonde et al., 2017).

The EULAR highlights that aerobic exercise is associated with improvements in pain and physical function (Macfarlane et al., 2017). A systematic review of high-quality clinical trials showed that aerobic exercise (eg., Nordic walking) reduced general fatigue and long-term physical fatigue (Garijo et al., 2022). It is important to highlight that The Borg CR-10 Scale can be used as an additional parameter for prescribing exercise intensity in aerobic training protocols for women with fibromyalgia, that is, the intensity of the physical exercise level is effective when the exercises are practiced in conjunction with the scale with this population (Andrade et al., 2017).

When compared to aerobic training, it is uncertain whether flexibility exercises improve outcomes such as quality of life, pain intensity, fatigue, stiffness and physical function because the quality of short- and long-term evidence is very low. This is due to studies with small samples and issues related to an unclear and high risk of bias (selection, performance and detection bias) (Kim et al., 2019). However, flexibility exercises are often incorporated into programs targeting individuals with fibromyalgia in the context of current practice, although, in some cases, they can be included in warmup and/or cool-down regimens, rather than being a stand-alone treatment intervention. In the scientific literature, flexibility exercises are being used in clinical trials as a control group, or as part of a relaxation intervention, which may further reinforce the lack of recognition of these exercises as a treatment in their own right (Kim et al., 2019).

Evidence suggests that moderate and moderate-to-high intensity resistance training improves muscle function, pain, sensitivity and strength in women with fibromyalgia; however, evidence quality is considered low. Recognizing that resistance training can improve well-being and symptoms may help promote this type of exercise as part of a balanced conditioning program for fibromyalgia patients and decrease fear-avoidance regarding possible increases in post-exercise muscle soreness. It is important to note that older (post-menopausal) women with fibromyalgia have the neuromuscular ability to gain strength, which may help improve and maintain functional mobility and reduce the risk of falls. A balanced conditioning program may also help reduce the risk of comorbidity and promote a more active lifestyle (Busch et al., 2013).

Aquatic training improves well-being and symptoms and may be a key factor in managing fibromyalgia (Bidonde et al., 2014). The movements performed in the aquatic environment, due to the heat and buoyancy of the water, relieve stress on the joints, being indicated for patients with chronic pain (López-Rodríguez et al., 2012). Aquatic physiotherapy, generally practiced in water heated between 32 and 33°C, is strongly indicated for the treatment of fibromyalgia. During immersion, sensory stimuli compete with painful stimuli, decreasing the pain cycle (Santos et al., 2020). Improvements in symptoms through aquatic exercise can be explained based on its vasodilating action, analgesia through the release of endorphins, increased capillarization, muscle trophism and oxygen consumption, in addition to a decrease in body weight. At the same time, the relaxation obtained with hot water reduces muscle contractures, helping to improve microcirculation (López-Rodríguez et al., 2012). In addition, this type of exercise improves the self-esteem of individuals with fibromyalgia (Santos et al., 2020).

Improvement in pain may be in part due to the water temperature, which provides immediate benefits for muscle aches or stiffness that often limit exercise tolerance on land. This decrease in symptoms can increase self-efficacy for exercise, mood, and sleep, among others, which may translate into an overall improvement in quality of life. Water exercises can make exercising more attractive, especially for unfit individuals. As such, exercise in warm water may be particularly beneficial as an initial exercise for sedentary individuals without exacerbating pain. The feeling of pleasure that arises from exercising in warm water may also improve adherence (Bidonde et al., 2014).

Another exercise option is the modified Pilates method, since it contains exercises to mobilize, stretch and strengthen muscles. Modified Pilates, currently the most widely used approach by physical therapists, is adapted to the practitioner and divided into levels of progression that emphasize the curvatures of the spine during exercises (Franco et al., 2019; Medeiros et al., 2020). Studies show that Pilates can be better than short-term home exercises and connective tissue massage for pain-pressure and anxiety thresholds (Ekici et al., 2017), but similar to yoga exercises (Palekar and Basu, 2014) and a control group without intervention (Komatsu et al., 2016); however, most of these are still pilot studies.

A systematic review of high-quality clinical trials provides moderate to strong evidence that active therapies, such as land and water aerobic exercise therapy, muscle strengthening, exergames, and other combinations of non-pharmacological treatments, improve long-term pain intensity, disability and physical performance. Short-term sleep function and quality, anxiety, and depression in individuals with fibromyalgia (Garijo et al., 2022).

Exercise programs have been consistently recommended for the management of fibromyalgia. Exercise is also one of the treatment approaches used in the clinical practice of physical therapists (Garrido-Ardila et al., 2021). Clinical trials indicate the benefits of exercise, especially in the form of aerobic exercise, resistance or flexibility training, and moderate-intensity resistance training, when compared to no exercise, has been shown to improve pain, sensitivity, physical functioning, and muscle strength. Aquatic exercise, defined as performing exercises in waist-deep or chest-level water, was also found to be effective in improving pain, stiffness, muscle strength and general well-being compared to no exercise; however, these results were similar in land exercises (Busch et al., 2007; Busch et al., 2013; Bidonde et al., 2014; Chinn et al., 2016).

Furthermore, dance-based training programs can be an effective intervention for people with fibromyalgia, leading to a significant reduction in pain level with an effect size that can be considered large. However, findings and conclusions from this meta-analysis should be interpreted with caution due to the small number of articles and considerable heterogeneity (Murillo-García et al., 2018). In this regard, more controlled studies are needed to investigate the effectiveness of these complementary methods to help treatment providers provide evidence-based fibromyalgia treatment recommendations (Verkamp et al., 2013; Alciati et al., 2021).

Exercise is effective for fibromyalgia along with cognitive behavioral therapy, education in coping strategies, and non-pharmacological interventions. Physiotherapists are prepared to promote safe and healthy physical activity, and this knowledge is necessary to propose strategies that support individuals with fibromyalgia to become physically active and maintain physical activity habits (Larsson et al., 2020).

#### 3.1.2 Manual therapy

Manual therapy has been defined in different styles. One refers to the manipulation of soft tissue and joints and another to the systematic mapping of soft tissue with rhythmic pressure to prevent, develop, maintain, rehabilitate or increase physical function or relieve pain. In physiotherapy practice, manual therapy plays an important role in the treatment of patients with musculoskeletal disorders, such as chronic back pain, migraines, anxiety, hypertension, depression and many other physical and psychological conditions that have been shown to respond positively to manual therapy. Connective tissue massage, an important element of manual therapy, deals with the skin and subcutaneous tissue. However, most literature reports show the benefits of manual therapy in healthy people, with few studies addressing these effects in individuals with fibromyalgia (Bervoets et al., 2015; Nadal-Nicolás et al., 2020).

Massage is the therapeutic modality used by 75% of individuals with fibromyalgia; however, only moderate evidence supports this intervention, since it can be extremely painful, although many patients still prefer it due to its potential long-term benefits. Massage intensity should be moderate to be beneficial and avoid pain (Bervoets et al., 2015; Nadal-Nicolás et al., 2020).

Massage therapy has been widely used for fibromyalgia as a complementary and alternative treatment. It can improve pain, anxiety, depression and sleep disturbances by the complex interplay of physical and mental modes of action (Imamura et al., 2012). A systematic review showed that massage therapy lasting ≥5 weeks provided immediate beneficial effects in improving pain, anxiety and depression and should considered a treatment for fibromyalgia (Li et al., 2014). In addition, a manual therapy protocol was effective in improving pain intensity, pressure pain sensitivity, the impact of fibromyalgia symptoms, sleep quality, and depressive symptoms (Castro-Sánchez et al., 2014).

Myofascial release has been used for individuals with fibromyalgia because ascending nociceptive pathways possibly involved in the central sensitization process, modulating the pain experience. However, there is uncertainty about its effectiveness compared to other interventions, given that manual therapy still exhibits non-specific effects. A systematic review investigated the effectiveness of manual therapy on pain, disease impact, and quality of life in individuals with fibromyalgia compared to a control group or other treatments. The results for fibromyalgia treatment were inconclusive and insufficient to support and recommend this intervention due to low-to-moderate evidence quality. To date, myofascial release has been the most widely used modality, but only general osteopathic treatment achieved a clinically relevant improvement in pain when compared to controls (Schulze et al., 2020).

Myofascial release is an emerging treatment for various conditions, with evidence regarding its symptom-relieving mechanisms. There is limited evidence on the effects of this type of intervention on fibromyalgia symptoms, with a systematic review showing moderate evidence for its effect on improving pain, sleep, and quality of life. However, more high-quality RCTs should be conducted in different geographic locations to generalize the findings (Ughreja et al., 2021).

In order to verify the effectiveness of different styles of massage therapy on fibromyalgia, meta-analysis showed that myofascial release had substantial positive effects on pain and medium effects on anxiety and depression at the end of treatment, in contrast to a placebo; the effects on pain and depression were maintained in the medium and short term, respectively. Narrative analysis suggests that myofascial release also improves fatigue, stiffness and quality of life; connective tissue massage improves depression and quality of life; manual lymphatic drainage is superior to connective tissue massage for stiffness, depression, and quality of life; Shiatsu improves pain, pressure pain threshold, fatigue, sleep and quality of life; and that Swedish massage provides no improvement. There is moderate evidence that myofascial release is beneficial for fibromyalgia symptoms. Limited evidence supports the application of connective tissue massage and Shiatsu. Manual lymphatic drainage may be superior to connective tissue massage and Swedish massage may be ineffective. Overall, most styles of massage consistently improved the quality of life of fibromyalgia patients (Yuan et al., 2015).

#### 3.1.3 Electrophysical agents and integrative and complementary therapie

Electrotherapeutic modalities are recommended as part of a fibromyalgia therapy program, according to the clinical status and general condition of the patient (Evcik et al., 2019). A systematic review with meta-analysis that evaluated the role of electrical stimulation in fibromyalgia (acts through the activation of descending inhibitory pathways, from the midbrain and brainstem, to inhibit the excitability of nociceptive neurons in the spinal cord, thus reducing the pain), combined or not with other types of therapies, concluded that this type of intervention significantly reduced pain; however, there was no significant difference in quality of life and fatigue and the evidence was considered low quality. When only transcutaneous electrical nerve stimulation was applied, no significant pain reduction was observed (Salazar et al., 2017).

The use of interferential current provided no significant improvement in pain relief for individuals with fibromyalgia. Ultrasound has been shown to reduce pain, stiffness and tender points, as well as improve physical function and sleep. Although low-level laser therapy has been suggested for pain relief by creating photochemical reactions that alter neuronal activity, its use in fibromyalgia is still controversial. There are studies indicating that pulsed electromagnetic fields and repetitive transcranial magnetic stimulation reduce pain and/or improve the quality of life of individuals with fibromyalgia (Evcik et al., 2019).

Repetitive transcranial magnetic stimulation is a non-invasive brain stimulation therapy commonly used to treat this disease. However, controversy over its effectiveness remains and a meta-analysis revealed that transcranial magnetic stimulation relieved pain and improved the quality of life of fibromyalgia patients, but based on current reports, it did not improve anxiety, depression or other symptoms (Sun et al., 2022).

Photobiomodulation therapy refers to the application of light to a biological system capable of inducing a photochemical process, mainly in mitochondria, with stimulation of energy production in the form of adenosine triphosphate, which can increase cellular metabolism and produce effects such as analgesia and regeneration. Tissue, reduction of muscle fatigue, among others (Bacelete and Gama, 2021). Exercise training and phototherapy (low-level laser therapy with light-emitting diode) are two of the approaches used to treat pain (Silva et al., 2015). A clinical trial developed by Silva et al. (2018) provided new insights into photobiomodulation therapy and physical exercise to improve pain in fibromyalgia patients. However, a more substantial effect was observed when combining photobiomodulation therapy with phasic exercise and these clinical effects contributed to improving pain, sleep disturbances and quality of life in individuals with fibromyalgia.

A review aimed to identify the efficacy and safety of electrotherapy in fibromyalgia treatment and showed that transcutaneous electrical nerve stimulation, non-invasive brain stimulation (transcranial direct current/magnetic stimulation) and light amplification by stimulated emission of radiation (LASER) were the most commonly used electrotherapy techniques for this condition. Currently, there is increasing data on the effectiveness of electrotherapy in managing fibromyalgia-related pain. In addition, non-invasive electrotherapy techniques are related to no or mild side effects. More studies are needed to identify optimal treatment protocols for each electrotherapy modality. Overall, the growing amount of data support the role of electrotherapy modalities and given the efficacy and safety profile, this intervention method may be included in fibromyalgia treatment protocols (Benlidayi, 2020).

In recent years, there has been an increase in complementary therapies to help manage rheumatic diseases. Evidence shows that some complementary therapy modalities (eg., acupuncture, diet, herbal medicine, homeopathy, massage, and supplements) are promising for the treatment of pain associated with various musculoskeletal conditions. As such, complementary therapies have gained increasing popularity, particularly among individuals with fibromyalgia for whom traditional medicine has been ineffective in relieving symptoms. Indeed, people with chronic health conditions such as fibromyalgia are associated with the widespread use of complementary therapies because they often provide temporary relief from some symptoms (Saad and de Medeiros, 2013; Ablin et al., 2015; Langhorst et al., 2017).

Systematic reviews evaluating the effectiveness of complementary therapies in fibromyalgia reveal beneficial effects arising from some modalities, while others showed a bias in favor of more conventional treatment, with no robust evidence (Saad and de Medeiros, 2013). A study by Mohabbat et al. (2019) on the use of complementary and integrative therapies in fibromyalgia patients showed that 98.1% of individuals reported using some of these interventions. The most widely used therapies were spiritual healing, massage therapy, chiropractic treatments, aromatherapy, exercises for a specific medical condition, as well as the ingestion of melatonin, magnesium, green tea and fish oil. Given the growing use of complementary and integrative medicine, healthcare providers should be aware of these various modalities and consider incorporating these practices into a multifaceted fibromyalgia treatment protocol (Bazzichi et al., 2020).

#### 3.1.4 Health education

The revised EULAR recommendations for fibromyalgia treatment indicate that the initial strategy should focus on patient education and nonpharmacological interventions (Macfarlane et al., 2017). Education is key to global health promotion and must equip people to take control and responsibility for their own health and that of their environment, and prepare them for empowerment, decision-making, participation, social control and acting on the conditions and determinants of their health and quality of life (Antunes et al., 2021a).

The chronic nature of fibromyalgia means that individuals often view the syndrome and its consequences in a negative light. To address this problem, interventions and programs that are solely educational or associated with physical exercise have been developed to promote the health of people with this condition (Antunes et al., 2021a). These programs aim at optimizing care through patient guidance provided by several professionals from different areas of knowledge, with advice on how to control pain and deal with lifestyle-associated problems (García-Ríos et al., 2019).

Patient education also allows individuals diagnosed with fibromyalgia to actively participate in their treatment, providing substantial personal benefits and encouraging the adoption of behaviors that will lead to biopsychosocial well-being and the best possible quality of life. Thus, patient education techniques focus on the idea that the patient is not facing a life-threatening illness, thereby helping to assure them that fibromyalgia is a real disease and recognizing its legitimacy (García-Ríos et al., 2019). Likewise, patient education should be based on written material or any other format that provides information about the nature of this disorder, as well as treatment, management strategies, and expected outcomes (Hawkins, 2013; Häuser et al., 2017).

Based on the premise that a better understanding of the nature of their illness results in better patient outcomes, education provided to fibromyalgia patients should specifically address the neuroscience of pain and central sensitization. Pain neuroscience education aims to reconceptualize pain and change inadequate cognitions of pain, attitudes toward pain, pain relief, disability, and physical dysfunction (Louw et al., 2011).

In this respect, growing evidence supports the use of in-person education in pain neuroscience for the treatment of individuals with chronic pain, especially fibromyalgia. A study by Pernambuco et al. (2019) conducted an educational intervention for fibromyalgia patients in the form of lectures and group activities. The most widely discussed topics in the 11-week interdisciplinary health education program were general information about fibromyalgia, body practices, physical activities and pharmacological approaches. Based on the results obtained at the end of the intervention, the authors suggested that health education can induce subjective and objective changes (immunological and neuroendocrine), which partially explains the health status of individuals with fibromyalgia.

Studies with distance-learning pain education are recent and of low quality. A clinical trial performed by Van Ittersum et al. (2014) to examine whether written education (booklet) on pain neuroscience improves disease perception, catastrophizing and health status in fibromyalgia patients, revealed that written pain neuroscience education exhibited slightly improved disease perception, but no clinically significant effects on pain, catastrophizing or daily activities. Thus, in-person pain neuroscience education sessions are necessary to alter inadequate cognitions and perceived health in individuals with fibromyalgia.

A systematic review of clinical trials found that patient education alone was not effective for pain, quality of life, or functionality in individuals with this condition. However, there is strong evidence of the effectiveness of combining patient education with exercise and active strategies to manage pain, quality of life and functionality in the short, medium and long term in individuals with fibromyalgia (Elizagaray-Garcia et al., 2016).

In this respect, multicomponent therapy, including primarily psychological and exercise therapy, is effective with regard to key fibromyalgia symptoms, self-efficacy, and physical fitness (Musekamp et al., 2019). In a systematic review with meta-analysis, Saracoglu et al. (2022) investigated whether the inclusion of pain neuroscience education produces additional benefits in patients with fibromyalgia and showed that pain neuroscience education combined with other non-pharmacological treatments (including exercise therapy) can be effective in improving the condition. Functional, symptoms related to pain, anxiety and depression in these individuals. However, more studies are needed, especially regarding the optimal duration and frequency of neuroscience education sessions on fibromyalgia-related pain.

Physical exercise is one of the most investigated approaches to the management of fibromyalgia. In addition, education in pain neurophysiology helps to understand the pain condition leading to maladaptive pain cognitions and coping strategies for people with chronic pain. This association between interventions should be encouraged. A clinical trial by Ceballos-Laita et al. (2021) found that pain education combined with physical exercise therapy appears to be more effective than physical exercise alone for improving physical function in women with fibromyalgia in the short term.

A meta-analysis that sought to determine the effect of non-pharmacological treatment based on pain neuroscience education, therapeutic exercises, cognitive behavioral therapy and mindfulness in fibromyalgia patients revealed that including pain neuroscience education in non-pharmacological treatment can effectively improve functional status, pain-related symptoms, anxiety, and depression in individuals with this disorder (Serrat et al., 2021).

The use of pain neuroscience education in chronic pain treatment has resulted in positive effects. However, in the context of primary health care, there are few data on the applicability of this therapeutic approach. A clinical trial showed that participating in 6 weekly sessions and a review session (1 month later) with pain neuroscience education plus exercise for individuals with fibromyalgia compared to the usual (pharmacological) treatment was shown to be more feasible and effective in primary health care (Areso-Bóveda et al., 2022).

So far, we have highlighted the main studies that address the “pain neuroscience education”, as these are still the studies with the highest quality, as the others on health education in fibromyalgia still have many limitations and are of low quality, so we need to have caution when indicating these programs yet, but we believe that in the future, these studies with higher quality will be available in the scientific literature and can be applied in clinical practice.

In this respect, health professionals and public policy makers must consider the costs and effectiveness of health education programs. Although educational approaches are widely used in practice and the prevention of other health problems, the current supporting evidence on health education in fibromyalgia needs to be clearer. This evidence is essential for health professionals to better address health education with fibromyalgia patients (Antunes et al., 2021a).

## 4 Future perspectives

Fibromyalgia is a common and complex long-term pain condition. Despite advances in our understanding and treatment, individuals report inconsistent health care delivery and frustrating experiences in the healthcare system. Feeling disappointed, ignored or powerless about the healthcare system and its professionals is common and participation in care support can also be difficult due to the distance of the treatment facility (Doebl et al., 2020). Many individuals report a long and difficult path of suffering starting before the onset of the illness and the unfolding illness gradually becoming persistent and overwhelming. Since understanding the disease cannot be separated from a person’s life, humanized educational interventions for people living with fibromyalgia are encouraged (Mengshoel, 2022).

New treatment approaches without sufficient supporting evidence are not recommended for individuals with fibromyalgia (low level of evidence but strong recommendation). Recent studies in fibromyalgia include methods such as laser and taping (Vayvay et al., 2016), hyperbaric oxygen therapy (a therapeutic modality in which a patient is subjected to the inhalation of pure oxygen at a pressure greater than atmospheric pressure, inside a hermetically sealed chamber with rigid walls) (Efrati et al., 2015; Barilaro et al., 2017), optic nerve stimulation (neuromodulation treatment that has been applied for cases of intractable chronic headache) (Plazier et al., 2014), dietary bioactive compounds (have beneficial effects on health, conferring benefits for the promotion and maintenance of health, including reducing the risk of developing chronic degenerative diseases such as cancer, cardiovascular diseases, osteoporosis, inflammation and type II diabetes, among others) (Shen et al., 2022; however, clinical evidence of these methods remains insufficient (Okifuji et al., 2016; Evcik et al., 2019).

Furthermore, a health education program called “Amigos do Fibro” (Fibro Friends) was developed (Antunes et al., 2022a) and validated (Antunes et al., 2022b) to promote the health of individuals with fibromyalgia. A study that obtained the approval of 23 health professionals and 45 individuals with fibromyalgia showed that “Friends of Fibro” exhibited adequate content validity and internal consistency and is therefore a valid tool for health professionals with this target audience in primary health care, enabling them to act as health promoting agents. As a result, the effectiveness of the project is currently being assessed and the authors are excited about developing this innovative proposal in Brazil (Antunes et al., 2021b; Antunes et al., 2021c; Torres et al., 2021).

The use of information and communication technologies has also become a trend in fibromyalgia care. For example, interventions encouraging positive thinking in people with fibromyalgia supported by online web-based platforms are being developed. The use of automated short message services (SMS) has also been developed to provide professional support and encourage exercise in fibromyalgia patients (Quero et al., 2012; Baños et al., 2014; Botella et al., 2016; Molinari et al., 2017; Molinari et al., 2018).

Virtual reality technology that allows individuals to be included in a virtual environment similar to the real world through a computer and interact with it has shown promising results in managing pain, anxiety or mood states and reducing the impact of fibromyalgia in women (Cortés-Pérez et al., 2021). An app has also been developed to promote self-care as a mobile health resource complementary to physiotherapy in fibromyalgia management. One example is ProFibro, the first free mobile application in Brazilian Portuguese for fibromyalgia, which provides patient education through animation, self-monitoring, sleep strategies, exercise programs, gratitude activities, family adjustments and health tips through notifications (Yuan and Marques, 2018).

Regarding patients’ perspectives on the effectiveness of non-pharmacological treatments for fibromyalgia, a study developed by Taylor et al. (2019) who investigated patient-reported outcomes of the use and efficacy of pharmacological and non-pharmacological treatment with an assessment of acceptability, showed that in relation to “physical therapies”, the symptom most relieved by physical therapy reported by individuals with fibromyalgia was pain muscle. Based on participant ratings, the three non-pharmacological treatments identified as most effective were hydrotherapy, thermal pad/hot water bottle, and massage. Treatments in the aquatic environment were rated significantly more effective than other “physical therapies”, acupuncture and transcutaneous electrical nerve stimulation.

In addition, the aforementioned study showed that the increase or induction of pain during “physical therapies” was the most reported side effect. Hydrotherapy and massage had significantly lower reported side effect scores than “physical therapies”. Therefore, it is evident that physical therapists should aim to change maladaptive pain beliefs when patients believe that the pain experienced during physical therapy is harmful.

Many individuals with fibromyalgia are not aware of some non-pharmacological treatment possibilities, this indicates that patient education is important to highlight the variety of treatments available, particularly physical therapy modalities, allowing for informed and optimal treatment decision making with their patients healthcare professional (Taylor et al., 2019).

Finally, efforts have also been made to improve the training and knowledge of physical therapists about fibromyalgia and their interaction with the work environment. Treating individuals with fibromyalgia challenges physical therapists, mainly due to professional deficiencies. In this respect, there is a need to equip physical therapists to respond to the needs of their patients with greater competence and less ambivalence. In addition, healthcare professionals need to recognize the difficulty physical therapists face in providing treatment to individuals with fibromyalgia (Roitenberg and Shoshana, 2021).

## 5 Conclusion

Several options for physiotherapy interventions have shown therapeutic effects in fibromyalgia. Among the different exercise interventions, aerobic and resistance exercise are the most indicated and widely used. Since flexibility exercises still exhibit low evidence, it is suggested that they be included in the warm-up process of other therapies. Aquatic exercise has fewer side effects than other physical exercises and is more widely accepted among individuals with fibromyalgia due to the relaxing factor of water temperature. With respect to manual therapy, it is concluded that myofascial release, connective tissue massage, manual lymphatic drainage and Shiatsu produce good results since, in general, most therapeutic massage techniques consistently improve the quality of life of fibromyalgia patients. Electrophysical agents are also used in the clinical practice of physical therapists who treat individuals with this disease. Given its good efficacy and safety profile, electrotherapy can be added to treatment protocols. In recent years, the use of complementary therapies has increased and evidence shows that some (e.g., acupuncture, diet, herbal medicine, homeopathy, massage, supplements) are promising for pain management associated with several musculoskeletal conditions; however, more robust evidence is needed. Finally, health education is essential and recommended for individuals with fibromyalgia. This type of intervention should promote care optimization through guidance physical therapists provide to individuals, advising them on how to control pain and deal with problems associated with their lifestyle. As such, combining multiple therapies (including exercise) to devise an optimal treatment plan for different individuals would be an important developmental step for fibromyalgia treatment in the future. More good quality studies are needed to show the benefits of physiotherapeutic treatment for the management of fibromyalgia.

It is important to highlight that there are important guidelines that have been increasingly studying pharmacological and non-pharmacological interventions for fibromyalgia and our study is not intended to replace these recommendations, that is, our aim is to show an overview of physical therapy interventions that seek to improve the symptoms of fibromyalgia, as well as promoting the health of these individuals. In addition, we encourage researchers to carry out systematic reviews and meta-analyses to deepen the degrees of recommendations and levels of evidence on the topic. In addition, we would like to reinforce that researchers should always be aware of the guidelines for non-pharmacological treatments for fibromyalgia that are published by renowned institutions in the field of rheumatology, such as the EULAR.

We want to highlight in this article that our objective is to show and encourage that physical therapists can use several possibilities to treat patients with fibromyalgia and that there is a clear distinction between the level of evidence of each of the references that we highlighted in the study and that clinicians should be attentive and deepen the studies in each type of intervention, since the conclusions of the cited articles are not all enough out in fully proven results. A guideline, a systematic review and a randomized clinical trial are not at the same level of evidence and the weight of each of them in clinical decision-making is not the same. Therefore, we highlight this limitation so that clinicians, when deciding on interventions to be used with their patients, analyze these aspects.

## References

[B1] Ablin J. Fitzcharles M. A. Buskila D. Shir Y. Sommer C. Häuser W. (2015). Treatment of fibromyalgia syndrome: Recommendations of recent evidence-based interdisciplinary guidelines with special emphasis on complementary and alternative therapies. Evid. Based. Complement. Altern. Med. 13, 485272–485277. 10.1155/2013/485272 PMC3856149 24348701

[B2] Alciati A. Nucera V. Masala I. F. Giallanza M. La Corte L. Giorgi V. (2021). One year in review 2021: Fibromyalgia. Clin. Exp. Rheumatol. 39 (3), S3–S12. 10.55563/clinexprheumatol/gz4i3i 34001307

[B3] Andrade C. P. Zamunér A. R. Forti M. França T. F. D. Silva E. D. (2017). The Borg CR-10 scale is suitable to quantify aerobic exercise intensity in women with fibromyalgia syndrome. Fisioter. Pesqui. 24 (3), 267–272. 10.1590/1809-2950/16558824032017

[B4] Antunes M. D. Couto L. A. Bertolini S. M. M. G. da Rocha Loures F. C. N. Schmitt A. C. B. Marques A. P. (2021). Effectiveness of interdisciplinary health education programs for individuals with fibromyalgia: A systematic review. J. Educ. Health Promot. 10, 64. 10.4103/jehp.jehp_592_20 34084811 PMC8057193

[B89] Antunes M. D. Schmitt A. C. B. Marques A. P. (2022a). Amigos de fibro (fibro friends): development of an educational program for the health promotion of fibromyalgia patients. Primary Health Care Research & Development 23 (e44), 1–7. 10.1017/S1463423621000773 PMC9381164 35924710

[B5] Antunes M. D. Schmitt A. C. B. Marques A. P. (2022b). Amigos de Fibro (fibro friends): Validation of an educational program to promote health in fibromyalgia. Int. J. Environ. Res. Public Health 19 (9), 5297. 10.3390/ijerph19095297 35564691 PMC9102409

[B6] Antunes M. Schmitt A. C. Marques A. (2021). Amigos de fibro (fibro friends): Development of A multidisciplinary health promotion program for fibromyalgia in Brazil. Jcr-Journal Clin. Rheumatology 27, s112. 10.1097/RHU.0000000000001781

[B7] Antunes M. Schmitt A. Marques A. P. (2021). AB0912-HPR AMIGOS de fibro (fibro friends): Educational program to promote the health of people with fibromyalgia in Brazil. Ann. Rheum. Dis. 80, 1478. 10.1136/annrheumdis-2021-eular.2598

[B8] Areso-Bóveda P. B. Mambrillas-Varela J. García-Gómez B. Moscosio-Cuevas J. I. González-Lama J. Arnaiz-Rodríguez E. (2022). Effectiveness of a group intervention using pain neuroscience education and exercise in women with fibromyalgia: A pragmatic controlled study in primary care. BMC Musculoskelet. Disord. 23 (1), 323–332. 10.1186/s12891-022-05284-y 35379222 PMC8978762

[B9] Bacelete V. S. B. Gama A. C. C. (2021). Therapeutic effects of photobiomodulation in the speech-language-hearing clinic: An integrative literature review. Rev. CEFAC 23 (1), e9120. 10.1590/1982-0216/20212319120

[B10] Baños R. M. Etchemendy E. Farfallini L. García-Palacios A. Quero S. Botella C. (2014). EARTH of well-being system: A pilot study of an information and communication technology-based positive psychology intervention. J. Posit. Psychol. 9 (6), 482–488. 10.1080/17439760.2014.927906

[B11] Barilaro G. Francesco Masala I. Parracchini R. Iesu C. Caddia G. Sarzi-Puttini P. (2017). The role of hyperbaric oxygen therapy in orthopedics and rheumatological diseases. Isr. Med. Assoc. J. 19 (7), 429–434. 28786258

[B12] Bazzichi L. Giacomelli C. Consensi A. Giorgi V. Batticciotto A. Di Franco M. (2020). One year in review 2020: Fibromyalgia. Clin. Exp. Rheumatol. 38 (123), S3–S8. 32116216

[B13] Benlidayi I. C. (2020). The effectiveness and safety of electrotherapy in the management of fibromyalgia. Rheumatol. Int. 40 (10), 1571–1580. 10.1007/s00296-020-04618-0 32524302

[B14] Bervoets D. C. Luijsterburg P. A. Alessie J. J. Buijs M. J. Verhagen A. P. (2015). Massage therapy has short-term benefits for people with common musculoskeletal disorders compared to no treatment: A systematic review. J. Physiother. 61 (3), 106–116. 10.1016/j.jphys.2015.05.018 26093806

[B15] Bidonde J. Busch A. J. Schachter C. L. Overend T. J. Kim S. Y. Góes S. M. (2017). Aerobic exercise training for adults with fibromyalgia. Cochrane Database Syst. Rev. 6 (6), CD012700. 10.1002/14651858.CD012700 28636204 PMC6481524

[B16] Bidonde J. Busch A. J. Webber S. C. Schachter C. L. Danyliw A. Overend T. J. (2014). Aquatic exercise training for fibromyalgia. Cochrane Database Syst. Rev. 2014 (10), CD011336. 10.1002/14651858.CD011336 PMC10638613 25350761

[B17] Borchers A. T. Gershwin M. E. (2015). Fibromyalgia: A critical and comprehensive review. Clin. Rev. Allergy Immunol. 49 (2), 100–151. 10.1007/s12016-015-8509-4 26445775

[B18] Botella C. Baños R. M. Etchemendy E. García-Palacios A. Alcañiz M. (2016). Psychological countermeasures in manned space missions:“EARTH” system for the Mars-500 project. Comput. Hum. Behav. 55, 898–908. 10.1016/j.chb.2015.10.010

[B19] Busch A. J. Barber K. A. Overend T. J. Peloso P. M. J. Schachter C. L. (2007). Exercise for treating fibromyalgia syndrome. Cochrane Database Syst. Rev. 2007 (4), CD003786. 10.1002/14651858.CD003786.pub2 PMC12832165 17943797

[B20] Busch A. J. Webber S. C. Richards R. S. Bidonde J. Schachter C. L. Schafer L. A. (2013). Resistance exercise training for fibromyalgia. Cochrane Database Syst. Rev. 2013 (12), CD010884. 10.1002/14651858.CD010884 PMC6544808 24362925

[B21] Castro-Sánchez A. M. Aguilar-Ferrándiz M. E. Matarán-Peñarrocha G. A. Sanchez-Joya M. D. M. Arroyo-Morales M. Fernandez-de-las-Penas C. (2014). Short-term effects of a manual therapy protocol on pain, physical function, quality of sleep, depressive symptoms, and pressure sensitivity in women and men with fibromyalgia syndrome: A randomized controlled trial. Clin. J. Pain 30 (7), 589–597. 10.1097/AJP.0000000000000008 24281285

[B22] Ceballos-Laita L. Mingo-Gómez M. T. Estébanez-de-Miguel E. Bueno-Gracia E. Navas-Cámara F. J. Verde-Rello Z. (2021). Does the addition of pain neurophysiology education to a therapeutic exercise program improve physical function in women with fibromyalgia syndrome? Secondary analysis of a randomized controlled trial. J. Clin. Med. 10 (11), 2518. 10.3390/jcm10112518 34200137 PMC8201111

[B23] Chinn S. Caldwell W. Gritsenko K. (2016). Fibromyalgia pathogenesis and treatment options update. Curr. Pain Headache Rep. 20 (4), 25–10. 10.1007/s11916-016-0556-x 26922414

[B24] Cortés-Pérez I. Zagalaz-Anula N. Ibancos-Losada M. D. R. Nieto-Escámez F. A. Obrero-Gaitán E. Osuna-Pérez M. C. (2021). Virtual reality-based therapy reduces the disabling impact of fibromyalgia syndrome in women: Systematic review with meta-analysis of randomized controlled trials. J. Pers. Med. 11 (11), 1167. 10.3390/jpm11111167 34834518 PMC8621064

[B25] Doebl S. Macfarlane G. J. Hollick R. J. (2020). No one wants to look after the fibro patient”. Understanding models, and patient perspectives, of care for fibromyalgia: Reviews of current evidence. Pain 161 (8), 1716–1725. 10.1097/j.pain.0000000000001870 32701832

[B26] Efrati S. Golan H. Bechor Y. Faran Y. Daphna-Tekoah S. Sekler G. (2015). Hyperbaric oxygen therapy can diminish fibromyalgia syndrome–prospective clinical trial. PloS one 10 (5), e0127012. 10.1371/journal.pone.0127012 26010952 PMC4444341

[B27] Ekici G. Unal E. Akbayrak T. Vardar-Yagli N. Yakut Y. Karabulut E. (2017). Effects of active/passive interventions on pain, anxiety, and quality of life in women with fibromyalgia: Randomized controlled pilot trial. Women Health 57 (1), 88–107. 10.1080/03630242.2016.1153017 26882533

[B28] Elizagaray-Garcia I. Muriente-Gonzalez J. Gil-Martinez A. (2016). Education for patients with fibromyalgia. A systematic review of randomised clinical trials. Rev. Neurol. 62 (2), 49–60. 10.33588/rn.6202.2015446 26758351

[B29] Evcik D. Ketenci A. Sindel D. (2019). The Turkish Society of Physical Medicine and Rehabilitation (TSPMR) guideline recommendations for the management of fibromyalgia syndrome. Turk. J. Phys. Med. Rehabil. 65 (2), 111–123. 10.5606/tftrd.2019.4815 31453551 PMC6706830

[B30] Fitzcharles M. A. Ste-Marie P. A. Goldenberg D. L. Pereira J. X. Abbey S. Choinière M. (2013). 2012 Canadian guidelines for the diagnosis and management of fibromyalgia syndrome: Executive summary. Pain Res. Manag. 18 (3), 119–126. 10.1155/2013/918216 23748251 PMC3673928

[B31] Franco K. F. M. Franco Y. R. D. S. Salvador E. M. E. S. do Nascimento B. C. B. Miyamoto G. C. Cabral C. M. N. (2019). Effectiveness and cost-effectiveness of the modified pilates method versus aerobic exercise in the treatment of patients with fibromyalgia: Protocol for a randomized controlled trial. BMC Rheumatol. 3 (1), 2–9. 10.1186/s41927-018-0051-6 PMC6390629 30886990

[B32] García-Ríos M. C. Navarro-Ledesma S. Tapia-Haro R. M. Toledano-Moreno S. Casas-Barragán A. Correa-Rodríguez M. (2019). Effectiveness of health education in patients with fibromyalgia: A systematic review. Eur. J. Phys. Rehabil. Med. 55 (2), 301–313. 10.23736/S1973-9087.19.05524-2 30698402

[B33] Garijo I. H. Del Barrio S. J. Gómez T. M. de la Fuente R. M. Laita L. C. (2022). Effectiveness of non-pharmacological conservative therapies in adults with fibromyalgia: A systematic review of high-quality clinical trials. J. Back Musculoskelet. Rehabil. 35 (1), 3–20. 10.3233/BMR-200282 34180405

[B34] Garrido-Ardila E. M. González-López-Arza M. V. Jiménez-Palomares M. García-Nogales A. Rodríguez-Mansilla J. (2021). Effects of physiotherapy vs. acupuncture in quality of life, pain, stiffness, difficulty to work and depression of women with fibromyalgia: A randomized controlled trial. J. Clin. Med. 10 (17), 3765. 10.3390/jcm10173765 34501213 PMC8432086

[B35] Häuser W. Ablin J. Perrot S. Fitzcharles M. A. (2017). Management of fibromyalgia: Practical guides from recent evidence-based guidelines. Pol. Arch. Intern. Med. 127, 47–56. 10.20452/pamw.3877 28075425

[B36] Hawkins R. A. (2013). Fibromyalgia: A clinical update. J. Am. Osteopath. Assoc. 113, 680–689. 10.7556/jaoa.2013.034 24005088

[B37] Imamura M. Furlan A. D. Dryden T. Irvin E. L. (2012). “Massage therapy,” in Evidence- based management of low back pain. Editors Dagenais S. Haldeman S. (Missouri: Elsevier), 216–228.

[B88] Kim S. Y. Busch A. J. Overend T. J. Schachter C. L. van der Spuy I. Boden C. (2019). Flexibility exercise training for adults with fibromyalgia. Cochrane Database Syst. Rev. 9, 1–93. 10.1002/14651858.CD013419 PMC6718217 31476271

[B38] Komatsu M. Avila M. A. Colombo M. M. Gramani-Say K. Driusso P. (2016). Pilates training improves pain and quality of life of women with fibromyalgia syndrome. Rev. Dor 17 (4), 274–278. 10.5935/1806-0013.20160088

[B39] Kundakci B. Kaur J. Goh S. L. Hall M. Doherty M. Zhang W. (2021). Efficacy of nonpharmacological interventions for individual features of fibromyalgia: A systematic review and meta-analysis of randomised controlled trials. Pain 163, 1432–1445. 10.1097/j.pain.0000000000002500 34813518

[B40] Langhorst J. Heldmann P. Henningsen P. Kopke K. Krumbein L. Lucius H. (2017). Complementary and alternative procedures for fibromyalgia syndrome: Updated guidelines 2017 and overview of systematic review articles. Schmerz (Berlin, Ger. 31 (3), 289–295. 10.1007/s00482-017-0206-1 28493227

[B41] Larsson A. Feldthusen C. Mannerkorpi K. (2020). Factors promoting physical activity in women with fibromyalgia: A qualitative interview study. BMJ open 10 (8), e031693. 10.1136/bmjopen-2019-031693 PMC7418681 32784252

[B42] Li Y. H. Wang F. Y. Feng C. Q. Yang X. F. Sun Y. H. (2014). Massage therapy for fibromyalgia: A systematic review and meta-analysis of randomized controlled trials. PloS one 9 (2), e89304. 10.1371/journal.pone.0089304 24586677 PMC3930706

[B43] López-Rodríguez M. M. Castro-Sánchez A. M. Fernández-Martínez M. Matarán-Penarrocha G. A. Rodríguez-Ferrer M. E. (2012). [Comparison between aquatic-biodanza and stretching for improving quality of life and pain in patients with fibromyalgia]. Aten. Primaria 44 (11), 641–649. 10.1016/j.aprim.2012.03.002 22591551 PMC7025635

[B44] Louw A. Diener I. Butler D. S. Puentedura E. J. (2011). The effect of neuroscience education on pain, disability, anxiety, and stress in chronic musculoskeletal pain. Arch. Phys. Med. Rehabil. 92 (12), 2041–2056. 10.1016/j.apmr.2011.07.198 22133255

[B45] Macfarlane G. J. Kronisch C. Dean L. E. Atzeni F. Häuser W. Fluß E. (2017). EULAR revised recommendations for the management of fibromyalgia. Ann. Rheum. Dis. 76 (2), 318–328. 10.1136/annrheumdis-2016-209724 27377815

[B46] Marques A. P. Santo A. D. S. D. E. Berssaneti A. A. Matsutani L. A. Yuan S. L. K. (2017). Prevalence of fibromyalgia: Literature review update. Rev. Bras. Reumatol. Engl. Ed. 57, 356–363. 10.1016/j.rbre.2017.01.005 28743363

[B47] Mascarenhas R. O. Souza M. B. Oliveira M. X. Lacerda A. C. Mendonça V. A. Henschke N. (2021). Association of therapies with reduced pain and improved quality of life in patients with fibromyalgia: A systematic review and meta-analysis. JAMA Intern. Med. 181 (1), 104–112. 10.1001/jamainternmed.2020.5651 33104162 PMC7589080

[B48] Medeiros S. A. D. Silva H. J. D. A. Nascimento R. M. D. Maia J. B. D. S. Lins C. A. D. A. Souza M. C. D. (2020). Mat pilates is as effective as aquatic aerobic exercise in treating women with fibromyalgia: A clinical, randomized and blind trial. Adv. Rheumatol. 60, 21. 10.1186/s42358-020-0124-2 32252822

[B49] Melo G. A. Araújo G. L. Vasconcelos A. M. Torro N. (2020). Recursos terapêuticos para a fibromialgia: uma revisão sistemática. Rev. Cont. Saude 20 (38), 49–56. 10.21527/2176-7114.2020.38.49-56

[B50] Mengshoel A. M. (2022). A long, winding trajectory of suffering with no definite start and uncertain future prospects–narratives of individuals recently diagnosed with fibromyalgia. Int. J. Qual. Stud. Health Well-being 17 (1), 2056956. 10.1080/17482631.2022.2056956 35356859 PMC8979520

[B51] Mohabbat A. B. Mahapatra S. Jenkins S. M. Bauer B. A. Vincent A. Wahner-Roedler D. L. (2019). Use of complementary and integrative therapies by fibromyalgia patients: A 14-year follow-up study. Mayo Clin. Proc. Innov. Qual. Outcomes 3 (4), 418–428. 10.1016/j.mayocpiqo.2019.07.003 31993560 PMC6978595

[B52] Molinari G. Roig Á. E. Herrero R. Comella N. F. L. Botella C. García-Palacios A. (2017). Development and pilot testing of a positive imagery intervention with online support in the treatment of fibromyalgia. Rev. Argent. Clin. Psic 6, 85. 10.24205/03276716.2017.1031

[B53] Molinari G. Garcia-Palacios A. Enrique Á. Roca P. Fernandez-Llanio Comella N. Botella C. (2018). The power of visualization: Back to the future for pain management in fibromyalgia syndrome. Pain Med. 19 (7), 1451–1468. 10.1093/pm/pnx298 29294081

[B54] Murillo-García Á. Villafaina S. Adsuar J. C. Gusi N. Collado-Mateo D. (2018). Effects of dance on pain in patients with fibromyalgia: A systematic review and meta-analysis. Evid. Based. Complement. Altern. Med. 1. 8709748. 10.1155/2018/8709748 PMC6188768 30364046

[B55] Musekamp G. Gerlich C. Ehlebracht-König I. Dorn M. Höfter A. Tomiak C. (2019). Evaluation of a self-management patient education programme for fibromyalgia—Results of a cluster-RCT in inpatient rehabilitation. Health Educ. Res. 34 (2), 209–222. 10.1093/her/cyy055 30689860

[B56] Nadal-Nicolás Y. Rubio-Arias J. Á. Martínez-Olcina M. Reche-García C. Hernández-García M. Martínez-Rodríguez A. (2020). Effects of manual therapy on fatigue, pain, and psychological aspects in women with fibromyalgia. Int. J. Environ. Res. Public Health 17 (12), 4611. 10.3390/ijerph17124611 PMC7345776 32604939

[B57] Okifuji A. Gao J. Bokat C. Hare B. D. (2016). Management of fibromyalgia syndrome in 2016. Pain Manag. 6 (4), 383–400. 10.2217/pmt-2016-0006 27306300 PMC5066139

[B58] Palekar T. J. Basu S. O. U. M. I. K. (2014). Comparative study of pilates exercise verses yogasana in the treatment of fibromyalgia syndrome: A pilot study. Int. J. Pharm. Bio Sci. 5 (3), 410–420.

[B59] Pernambuco A. P. Carvalho L. D. S. C. Schetino L. P. L. Polese J. C. Viana R. D. S. Reis D. D. Á. (2019). Effects of a health education program on cytokines and cortisol levels in fibromyalgia patients: A randomized controlled trial. Adv. Rheumatol. 58 (1), 21. 10.1186/s42358-018-0022-z 30657084

[B60] Plazier M. Dekelver I. Vanneste S. Stassijns G. Menovsky T. Thimineur M. (2014). Occipital nerve stimulation in fibromyalgia: A double-blind placebo-controlled pilot study with a six-month follow-up. Neuromodulation 17 (3), 256–263. 10.1111/ner.12121 24118206

[B61] Quero S. Molés M. Pérez-Ara M. Á. Botella C. Baños R. M. (2012). An online emotional regulation system to deliver homework assignments for treating adjustment disorders. Stud. Health Technol. Inf. 181, 273–277. 22954870

[B62] Roitenberg N. Shoshana A. (2021). Physiotherapists’ accounts of fibromyalgia: Role-uncertainty and professional shortcomings. Disabil. Rehabil. 43 (4), 545–552. 10.1080/09638288.2019.1632939 31257947

[B63] Saad M. de Medeiros R. (2013). Complementary therapies for fibromyalgia syndrome–A rational approach. Curr. Pain Headache Rep. 17 (8), 354–358. 10.1007/s11916-013-0354-7 23801007

[B64] Salazar A. P. Stein C. Marchese R. R. Plentz R. D. Pagnussat A. S. (2017). Electric stimulation for pain relief in patients with fibromyalgia: A systematic review and meta-analysis of randomized controlled trials. Pain Physician 20 (2), 15–25. 10.36076/ppj/2017/25 28158150

[B65] Santos J. M. Ribeiro V. G. C. Flor J. Carvalho M. M. Vitorino D. F. M. Lacerda A. C. R. (2020). Fisioterapia aquática: Uma intervenção para mulheres com fibromialgia. Exp. Ext. 25 (2), 103–112. 10.15210/ee.v25i2.18158

[B66] Saracoglu I. Akin E. Aydin Dincer G. B. (2022). Efficacy of adding pain neuroscience education to a multimodal treatment in fibromyalgia: A systematic review and meta‐analysis. Int. J. Rheum. Dis. 25 (4), 394–404. 10.1111/1756-185X.14293 35061337

[B67] Sauch Valmaña G. Vidal-Alaball J. Poch P. R. Peña J. M. Panadés Zafra R. Cantero Gómez F. X. (2020). Effects of a physical exercise program on patients affected with fibromyalgia. J. Prim. Care Community Health 11, 2150132720965071. 10.1177/2150132720965071 33084477 PMC7786411

[B68] Schulze N. B. Salemi M. M. Alencar G. G. Moreira M. C. Diqueira G. R. (2020). Efficacy of manual therapy on pain, impact of disease, and quality of life in the treatment of fibromyalgia: A systematic review. Pain Physician 23, 461–475. 10.36076/ppj.2020/23/461 32967389

[B69] Serrat M. Sanabria-Mazo J. P. Almirall M. Musté M. Feliu-Soler A. Méndez-Ulrich J. L. (2021). Effectiveness of a multicomponent treatment based on pain neuroscience education, therapeutic exercise, cognitive behavioral therapy, and mindfulness in patients with fibromyalgia (fibrowalk study): A randomized controlled trial. Phys. Ther. 101 (12), pzab200. 10.1093/ptj/pzab200 34499174

[B70] Shen C. L. Schuck A. Tompkins C. Dunn D. M. Neugebauer V. (2022). Bioactive compounds for fibromyalgia-like symptoms: A narrative review and future perspectives. Int. J. Environ. Res. Public Health 19 (7), 4148. 10.3390/ijerph19074148 35409832 PMC8998198

[B71] Silva M. M. Albertini R. Carvalho P. T. C. Leal-Junior E. C. P. Bussadori S. K. Vieira S. S. (2018). Randomized, blinded, controlled trial on effectiveness of photobiomodulation therapy and exercise training in the fibromyalgia treatment. Lasers Med. Sci. 33 (2), 343–351. 10.1007/s10103-017-2388-2 29170901

[B72] Silva M. M. Albertini R. Leal-Junior E. C. P. Carvalho P. T. C. Silva J. A. Bussadori S. K. (2015). Effects of exercise training and photobiomodulation therapy (EXTRAPHOTO) on pain in women with fibromyalgia and temporomandibular disorder: Study protocol for a randomized controlled trial. Trials 16 (1), 252–258. 10.1186/s13063-015-0765-3 26040789 PMC4464876

[B73] Sun P. Fang L. Zhang J. Liu Y. Wang G. Qi R. (2022). Repetitive transcranial magnetic stimulation for patients with fibromyalgia: A systematic review with meta-analysis. Pain Med. 23 (3), 499–514. 10.1093/pm/pnab276 34542624

[B74] Taylor S. J. Steer M. Ashe S. C. Furness P. J. Haywood-Small S. Lawson K. (2019). Patients’ perspective of the effectiveness and acceptability of pharmacological and non-pharmacological treatments of fibromyalgia. Scand. J. Pain 19 (1), 167–181. 10.1515/sjpain-2018-0116 30315738

[B75] Torres S. Antunes M. Yuan S. Schmitt A. C. Marques A. (2021). Clinical and Experimental Rheumatology, 39, 218–219.Amigos de FIBRO (FIBRO friends): Development of a multidisciplinary health promotions program for individuals with fibromyalgia in Brazil Clin. Exper Rheumatology

[B76] Ughreja R. A. Venkatesan P. Gopalakrishna D. B. Singh Y. P. (2021). Effectiveness of myofascial release on pain, sleep, and quality of life in patients with fibromyalgia syndrome: A systematic review. Complement. Ther. Clin. Pract. 45, 101477. 10.1016/j.ctcp.2021.101477 34507243

[B77] Uruş G. Altuğ F. Cavlak U. (2021). Treatment preferences of physiotherapists in fibromyalgia syndrome and the related chronic pain: A descriptive study. Sports Med. Journal/Medicina Sport. 17 (2), 3336–3341.

[B78] Van Ittersum M. W. van Wilgen C. P. van der Schans C. P. Lambrecht L. Groothoff J. W. Nijs J. (2014). Written pain neuroscience education in fibromyalgia: A multicenter randomized controlled trial. Pain Pract. 14 (8), 689–700. 10.1111/papr.12137 24251724

[B79] Vayvay E. S. Tok D. Turgut E. Tunay V. B. (2016). The effect of laser and taping on pain, functional status and quality of life in patients with fibromyalgia syndrome: A placebo-randomized controlled clinical trial. J. Back Musculoskelet. Rehabil. 29 (1), 77–83. 10.3233/BMR-150600 26406218

[B80] Verkamp E. K. Flowers S. R. Lynch-Jordan A. M. Taylor J. Ting T. V. Kashikar-Zuck S. (2013). A survey of conventional and complementary therapies used by youth with juvenile-onset fibromyalgia. Pain Manag. Nurs. 14 (4), e244–e250. 10.1016/j.pmn.2012.02.002 24315277 PMC3857559

[B81] Wolfe F. Walitt B. T. Häuser W. (2014). What is fibromyalgia, how is it diagnosed, and what does it really mean? Arthritis Care Res. 66 (7), 969–971. 10.1002/acr.22207 24127234

[B82] Wolfe F. Clauw D. J. Fitzcharles M. A. Goldenberg D. L. Katz R. S. Mease P. (2010). The American College of Rheumatology preliminary diagnostic criteria for fibromyalgia and measurement of symptom severity. Arthritis Care Res. 62 (5), 600–610. 10.1002/acr.20140 20461783

[B83] Wolfe F. Clauw D. J. Fitzcharles M. A. Goldenberg D. L. Häuser W. Katz R. L. (2016). 2016 Revisions to the 2010/2011 fibromyalgia diagnostic criteria. Semin. Arthritis Rheum. 46 (3), 319–329. 10.1016/j.semarthrit.2016.08.012 27916278

[B84] Wolfe F. Clauw D. J. Fitzcharles M. A. Goldenberg D. L. Häuser W. Katz R. S. (2011). Fibromyalgia criteria and severity scales for clinical and epidemiological studies: A modification of the ACR preliminary diagnostic criteria for fibromyalgia. J. Rheumatol. 38 (6), 1113–1122. 10.3899/jrheum.100594 21285161

[B85] Wolfe F. Smythe H. A. Yunus M. B. Bennett R. M. Bombardier C. Goldenberg D. L. (1990). The American College of rheumatology 1990 criteria for the classification of fibromyalgia. Report of the multicenter criteria committee. Arthritis Rheum. 33 (2), 160–172. 10.1002/art.1780330203 2306288

[B86] Yuan S. L. K. Marques A. P. (2018). Development of ProFibro—A mobile application to promote self-care in patients with fibromyalgia. Physiotherapy 104 (3), 311–317. 10.1016/j.physio.2018.04.005 30025715

[B87] Yuan S. L. K. Matsutani L. A. Marques A. P. (2015). Effectiveness of different styles of massage therapy in fibromyalgia: A systematic review and meta-analysis. Man. Ther. 20 (2), 257–264. 10.1016/j.math.2014.09.003 25457196

